# “Beet-ing” the Mountain: A Review of the Physiological and Performance Effects of Dietary Nitrate Supplementation at Simulated and Terrestrial Altitude

**DOI:** 10.1007/s40279-017-0744-9

**Published:** 2017-06-02

**Authors:** Oliver Michael Shannon, Kerry McGawley, Linn Nybäck, Lauren Duckworth, Matthew John Barlow, David Woods, Mario Siervo, John Paul O’Hara

**Affiliations:** 1 Research Institute for Sport, Physical Activity, and Leisure, Leeds Beckett University, Leeds, LS6 3QS UK; 2 Swedish Winter Sports Research Centre, Department of Health Sciences, Mid Sweden University, Östersund, Sweden; 3 Defence Medical Services, Royal Centre for Defence Medicine, Birmingham, B152TH UK; 4 Institute of Cellular Medicine, University of Newcastle, Newcastle upon Tyne, NE45PL UK

## Abstract

Exposure to altitude results in multiple physiological consequences. These include, but are not limited to, a reduced maximal oxygen consumption, drop in arterial oxygen saturation, and increase in muscle metabolic perturbations at a fixed sub-maximal work rate. Exercise capacity during fixed work rate or incremental exercise and time-trial performance are also impaired at altitude relative to sea level. Recently, dietary nitrate (NO_3_^−^) supplementation has attracted considerable interest as a nutritional aid during altitude exposure. In this review, we summarise and critically evaluate the physiological and performance effects of dietary NO_3_^−^ supplementation during exposure to simulated and terrestrial altitude. Previous investigations at simulated altitude indicate that NO_3_^−^ supplementation may reduce the oxygen cost of exercise, elevate arterial and tissue oxygen saturation, improve muscle metabolic function, and enhance exercise capacity/performance. Conversely, current evidence suggests that NO_3_^−^ supplementation does not augment the training response at simulated altitude. Few studies have evaluated the effects of NO_3_^−^ at terrestrial altitude. Current evidence indicates potential improvements in endothelial function at terrestrial altitude following NO_3_^−^ supplementation. No effects of NO_3_^−^ supplementation have been observed on oxygen consumption or arterial oxygen saturation at terrestrial altitude, although further research is warranted. Limitations of the present body of literature are discussed, and directions for future research are provided.

## Key Points

**Table:** 

The findings of this review demonstrate that dietary nitrate (NO_3_^−^) supplementation may reduce the oxygen cost of exercise, elevate arterial and tissue oxygenation, improve muscle metabolic function, and enhance exercise capacity/performance at simulated altitude.
Current preliminary evidence from training studies conducted at simulated altitude suggests that NO_3_^−^ supplementation has no effect on performance-related adaptations, although further investigations are warranted.
Additional studies are required to confirm whether the beneficial effects of NO_3_^−^ supplementation that have been demonstrated at simulated altitude also manifest at terrestrial altitude.

## Introduction

Every year, millions of individuals ascend to altitude for recreational, occupational, and sporting purposes [1]. Reasons for ascending to altitude include, amongst others, tourism, hiking and mountaineering, skiing, military deployment, sports training camps, and athletic competition. With increasing altitude, there is a decrease in barometric pressure and a concomitant decline in the partial pressure of oxygen (PO_2_), such that the high-altitude environment is hypoxic relative to sea level (normoxia). Exposure to this hypoxic environment decreases the amount of oxygen (O_2_) reaching the lungs, blood, and ultimately tissue [2, 3]. Consequently, maximal O_2_ consumption (${\dot{\rm V}}{\rm O}_{2\hbox{max} }$) decreases [4, 5]. The absolute ${\dot{\rm V}}{\rm O}_{2}$ necessary to maintain a given sub-maximal work rate is typically the same at sea level and altitude up to ~4300 m, although it may be slightly reduced at higher altitudes (e.g., 5260 m) consequent to alterations in substrate oxidation [6]. Nevertheless, the relative percentage of ${\dot{\rm V}}{\rm O}_{2\hbox{max} }$ utilized is higher at altitude relative to sea level [7]. As such, muscle metabolic perturbations are increased [8–11]. Furthermore, exercise time to exhaustion (TTE) and time-trial (TT) performance are impaired at altitude compared with sea level [7]. Prolonged altitude exposure results in acclimatization, comprising multiple renal, cardio-pulmonary, and hematological adaptations that act to increase the delivery of O_2_ to the tissue [12]. However, these adaptive changes can take days or weeks to fully manifest, and sea-level exercise performance/capacity is never fully attained, even with prolonged acclimatization [7].

Nitric oxide (NO) is a pleiotropic signaling molecule and a regulator of multiple physiological processes, many of which are altered by hypoxia, including mitochondrial function [13, 14], cerebral and muscle tissue blood flow [15–17], muscle metabolism [10, 11], and endothelial function [18, 19]. NO plays a fundamental role in the response to hypoxia and has been implicated in hypoxia-induced vasodilation, a mechanism designed to ensure appropriate matching between O_2_ delivery and the metabolic demands of muscle and cerebral tissue when O_2_ availability is low [15, 20]. Elevated levels of NO [21], alongside hematological (e.g., erythropoiesis and increased hemoglobin mass) and ventilatory (e.g., increased hypoxic ventilatory response) changes [22], have been associated with successful adaptation and acclimatization to altitude in lowlanders and altitude residents. In particular, Tibetan highlanders who have adapted to the genetic selection pressures of living at high altitude, manifest greatly increased plasma and pulmonary NO bioavailability [18, 23, 24]. Such adaptations afford distinctive metabolic [25] and microcirculatory [18] benefits that translate into an extraordinary capacity to cope with the challenges of hypoxia relative to unacclimatized lowlanders. Conversely, prior to acclimatization, lowlanders exhibit suppressed NO bioavailability with altitude exposure, and both failure to acclimatize and high-altitude illness have been associated with insufficient NO generation in hypoxia [26–28].

**Fig. 1:**
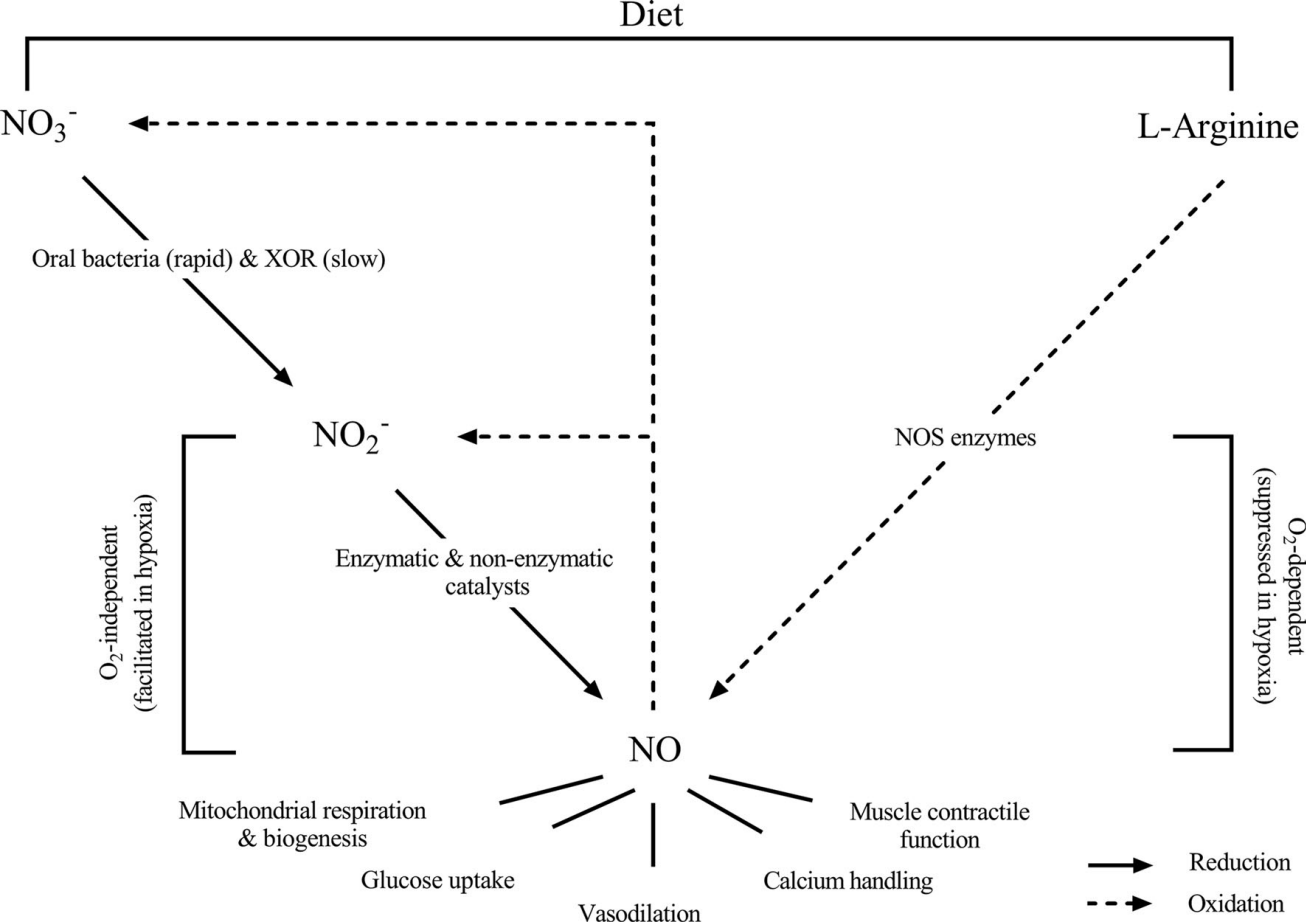
A schematic representation of the metabolic pathways for nitric oxide (NO) generation in the human body (adapted from Jones [32], with permission). The ‘traditional’ pathway for NO generation involves oxidation of the semi-essential amino acid l-arginine, in a reaction which requires the availability of O_2_ and is catalysed via the NO synthase (NOS) enzymes. This O_2_-dependent reaction is suppressed in hypoxia. Alternatively, NO can be generated via the nitrate (NO_3_^−^)–nitrite (NO_2_^−^)–NO pathway. Here, NO_3_^−^ from dietary sources or produced as an oxidation product of the l-arginine pathway is reduced into NO_2_^−^ via oral bacteria (a rapid pathway) or via the enzyme xanthine oxidoreductase (XOR) (a slower pathway). NO_2_^−^ can subsequently be reduced into NO via multiple catalysts, particularly under conditions of low O_2_ availability. This second pathway may serve to maintain or enhance NO signalling under conditions of hypoxia, such as that experienced at an altitude

Given the importance of NO during hypoxic exposure, it has been widely speculated that increasing NO bioavailability via dietary supplementation might confer beneficial effects in individuals ascending to altitude [29–32]. Endogenous NO production occurs via two distinct and uniquely different pathways that may be targeted to increase NO generation. First, NO can be produced via oxidation of the semi-essential amino acid l-arginine. This reaction is catalyzed by the NO synthase (NOS) enzymes and requires O_2_ as a co-substrate. In vitro evidence suggests that NO generated via the l-arginine NOS pathway is suppressed in hypoxia [33], although in vivo evidence is less clear [34–36]. Alternatively, NO can be generated via the reduction of nitrate (NO_3_^−^) and nitrite (NO_2_^−^) through the recently elucidated NO_3_^−^–NO_2_^−^–NO pathway [37]. Consumption of NO_3_^−^-rich foods such as leafy green vegetables and beetroot or NO_3_^−^-salts (e.g., sodium [Na]- or potassium [K^+^]-NO_3_^−^) rapidly and substantially elevates plasma [NO_3_^−^] (square brackets denote concentration) [38]. Exogenous NO_3_^−^ mixes with endogenously derived NO_3_^−^, which is an oxidation product of the l-arginine NOS pathway, in the blood and can be slowly reduced to NO_2_^−^ by the enzyme xanthine oxidoreductase [39]. A more rapid pathway for NO_3_^−^ reduction also exists, which is facilitated through recirculation of NO_3_^−^ into the mouth via the salivary glands [40]. Here, oral bacteria reduce NO_3_^−^ to NO_2_^−^ via reductase enzymes [41]. A portion of the bacterially generated NO_2_^−^ is transformed into NO in the stomach [42, 43]. However, the majority enters systemic circulation and may be further reduced to NO via enzymatic and non-enzymatic catalysts [37, 44] including, amongst others, xanthine oxidoreductase [45, 46], deoxygenated hemoglobin [47] and myoglobin [48], and cytochrome c oxidase (COX) [49]. Importantly, these pathways for NO generation via NO_2_^−^ reduction are all considerably enhanced in hypoxic conditions [37]. The NO_3_^−^–NO_2_^−^–NO pathway is therefore of particular importance in hypoxia and is viewed as an important “back-up” system for maintaining and/or enhancing NO bioavailability and signaling in this environment (Fig. 1). It should be noted that recent evidence suggests a possible direct role of NO_2_^−^ on skeletal muscle and mitochondrial function and in hypoxia-induced vasodilation [50]. Thus, any effects of NO_3_^−^ consumption might, at least in part, be related to NO_2_^−^-dependent signaling, rather than being solely attributable to NO [50].

Interestingly, recent evidence from studies conducted at sea level suggests that NO_3_^−^ supplementation can reduce resting blood pressure (BP) [51–53], lower the O_2_ cost of exercise [54–56], and augment both exercise capacity and performance [55, 57–59]. Logically, greater effects of NO_3_^−^ supplementation might be expected in hypoxia, given the particularly important role that NO plays in this environment and that NO production via the l-arginine NOS pathway is suppressed yet NO_2_^−^ reduction is enhanced in hypoxia. Furthermore, a reduced O_2_ cost of exercise is likely to be especially meaningful for individuals exercising in a low-O_2_ environment.

Although several recent articles have been published reviewing the potential benefits of dietary NO_3_^−^ supplementation at sea level [31, 32, 59–61], there is presently no review of the literature focusing on its specific applications for individuals exercising at altitude, despite a considerable and broad body of evidence emerging in the area. The purpose of the present review is to address this need by summarizing and critically evaluating the findings of recent research exploring the effects of NO_3_^−^ supplementation on cardiopulmonary and metabolic responses to exercise and exercise performance at altitude. The potential effects of NO_3_^−^ supplementation on adaptations to altitude training are also explored. Throughout the review, key limitations of the present body of evidence will be discussed and directions for future research suggested.

## Study Selection and Characteristics

Studies were identified for this review by searching online journals and the databases PubMed and SPORTDiscus. This review includes studies that assessed the effects of NO_3_^−^ supplementation during both acute (~1–5 h) and chronic (1–28 days) altitude exposure. We included studies conducted at terrestrial altitude, simulated altitude via normobaric hypoxia (i.e., the altitude PO_2_ is replicated by lowering the fraction of inspired O_2_ [F_I_O_2_] whilst maintaining sea-level barometric pressure), and hypobaric hypoxia (i.e., the altitude PO_2_ is replicated by lowering the barometric pressure whilst the F_I_O_2_ is unchanged from sea-level values). Terrestrial altitudes ranged from 1300 to 5300 m. Reported simulated altitudes were ~2500–5000 m (F_I_O_2_ 11–15.4%) in normobaric hypoxia and 3500 m in a hypobaric chamber. The dose of NO_3_^−^ ranged from ~5 to 15.2 mmol·day^−1^. Supplementation duration ranged from an acute dose 1.5–3 h pre-trial, to chronic loading for up to 11 days.

## Physiological Effects of Nitrate (NO_3_^−^) Supplementation in Hypoxia

### Oxygen (O_2_) Consumption During Steady-State Constant Work-Rate Exercise

Larsen et al. [54] first reported a reduction in steady-state ${\dot{\rm V}}{\rm O}_{2}$ consequent to NO_3_^−^ supplementation with ${\dot{\rm V}}{\rm O}_{2}$ ~5% lower during cycle ergometry exercise in normoxia. This effect was unexpected, as the ${\dot{\rm V}}{\rm O}_{2}$ requirement of a given workload is typically regarded as unchangeable, but has since been confirmed in multiple subsequent investigations in normoxia [38, 55–57, 62, 63].

Masschelein et al. [29] confirmed the O_2_-sparing effect of NO_3_^−^ supplementation during exercise in normobaric hypoxia. ${\dot{\rm V}}{\rm O}_{2}$ was ~4% lower during cycle ergometry exercise at 45% peak O_2_ uptake (${\dot{\rm V}}{\rm O}_{{2{\rm peak}}}$) in extreme normobaric hypoxia (F_I_O_2_ 11%, ~5000 m). Subsequent studies have reported ${\dot{\rm V}}{\rm O}_{2}$ reductions of 4–10% (similar magnitudes to those reported in normoxia) during cycle ergometry [29, 30, 36] and treadmill running exercise [64] across a range of simulated altitudes (F_I_O_2_ 11–15.4%, ~2500–5000 m). Interestingly, one study reported lower steady-state ${\dot{\rm V}}{\rm O}_{2}$ in normobaric hypoxia (F_I_O_2_ 13.1%, ~3500 m) but not normoxia [36] following NO_3_^−^ supplementation. This suggests that NO_3_^−^ supplementation might be especially effective at reducing the O_2_ cost of exercise in hypoxia, possibly due to greater conversion of NO_2_^−^ into NO and/or more effective modulation of O_2_-consuming cellular processes.

To the authors’ knowledge, only one study has reported the effects of NO_3_^−^ supplementation on ${\dot{\rm V}}{\rm O}_{2}$ during acute exercise in hypobaric hypoxia [65]. In contrast to some evidence obtained in normobaric hypoxia, NO_3_^−^ supplementation did not influence ${\dot{\rm V}}{\rm O}_{2}$ during cycle ergometry exercise in a hypobaric hypoxic chamber simulating 3500 m altitude. These inconsistent findings are puzzling but might be related to the high training status of the participants (${\dot{\rm V}}{\rm O}_{2\hbox{max} }$ at 1600 m altitude: ~61 ml·kg^−1^·min^−1^; see Sect. 4 for details of how training status may modulate the effects of NO_3_^−^ supplementation) rather than the exercise environment. Supporting this notion, three recent studies have reported no effect of NO_3_^−^ supplementation on ${\dot{\rm V}}{\rm O}_{2}$ during sub-maximal exercise in normobaric hypoxia (F_I_O_2_ 11–15.4%, ~2500–5000 m) in highly-trained individuals (${\dot{\rm V}}{\rm O}_{2\hbox{max} }$: >60 ml·kg^−1^·min^−1^) [66–68]. Moreover, a recent investigation at sea level reported a significant inverse relationship between ${\dot{\rm V}}{\rm O}_{{2{\rm peak}}}$ and the reduction in ${\dot{\rm V}}{\rm O}_{2}$ consequent to NO_3_^−^ supplementation [58]. However, direct comparison of the effects of NO_3_^−^ ingestion on ${\dot{\rm V}}{\rm O}_{2}$ in normobaric and hypobaric hypoxia is warranted to exclude the influence of other potentially confounding methodological variables.

The reduction in ${\dot{\rm V}}{\rm O}_{2}$ subsequent to NO_3_^−^ supplementation is unlikely to be related to a shift towards anaerobic adenosine triphosphate (ATP) generation, as blood [lactate] [54], muscle pH [69], and glycolytic ATP turnover [62] are typically unchanged following NO_3_^−^ ingestion. Instead, it has been suggested that the lower ${\dot{\rm V}}{\rm O}_{2}$ reported after NO_3_^−^ supplementation might be related to improvements in the efficiency of muscle force generation [62] and/or mitochondrial respiration [70].

In the study by Bailey et al. [62], NO_3_^−^ supplementation attenuated intramuscular phosphocreatine (PCr), adenosine diphosphate (ADP), and inorganic phosphate (P_i_) perturbations and lowered ${\dot{\rm V}}{\rm O}_{2}$ during leg-extension exercise in normoxia, suggesting a lower ATP cost of muscle force generation [62]. The ATP cost of muscle force production is primarily accounted for by cross-bridge cycling and calcium (Ca^2+^) handling [71, 72]. Given NO has been demonstrated to slow cross-bridge cycling kinetics [73, 74] and inhibit Ca^2+^-ATPase activity [75, 76], the lower ATP requirement of muscle force generation following NO_3_^−^ supplementation may be related to a NO-mediated reduction in the ATP cost of one or both of these processes [62]. Recently, Hernandez et al. [77] reported increased muscle force generation and elevated expression of the Ca^2+^ handling protein calsequestrin 1 and the dihydropyridine receptor in murine type II muscle fibers following NO_3_^−^ supplementation. It was proposed that similar effects in humans might allow muscle activation at a lower frequency for an equivalent force production, thus decreasing motor unit recruitment and lowering the ATP cost (and therefore improving the efficiency) of force production.

Larsen et al. [70] reported improvements in mitochondrial efficiency following NO_3_^−^ supplementation. Supplementation with NaNO_3_^−^ for 3 days increased the phosphate/O_2_ (P/O) ratio of mitochondria harvested from the vastus lateralis of healthy men, an effect that was significantly correlated with the reduced in vivo ${\dot{\rm V}}{\rm O}_{2}$. The authors reported reduced expression of the ATP/ADP translocase protein (ANT) and a tendency towards reduced expression of uncoupling protein 3 (UCP3). Such effects may have reduced proton leak during oxidative phosphorylation and could account for the improved P/O ratio and lower pulmonary ${\dot{\rm V}}{\rm O}_{2}$ [70]. It was proposed that these changes to protein expression may manifest as a consequence of NO competitively inhibiting O_2_ binding to COX [78], which may be detected by the mitochondria as hypoxia, activating signaling pathways that downregulate ANT [70]. Given greater inhibition of COX would be expected when PO_2_ is low, it is possible that this mechanism may be more active in hypoxia than in normoxia [61]. In contrast to the findings of Larsen et al. [70], Whitfield et al. [79] reported no effect of NO_3_^−^-rich beetroot juice on mitochondrial efficiency despite a reduced ${\dot{\rm V}}{\rm O}_{2}$. These disparate results may be related to the form of NO_3_^−^ administered (i.e., NaNO_3_^−^ vs. beetroot juice), although direct comparison is required to eliminate other methodological discrepancies [80]. Interestingly, one recent study [81] reported greater reductions in ${\dot{\rm V}}{\rm O}_{2}$ following beetroot juice versus NaNO_3_^−^ supplementation in normoxia, possibly as a consequence of the beetroot juice polyphenols and antioxidants enhancing NO generation, further suggesting different effects of these two NO_3_^−^-containing supplements.

### O_2_ Uptake Kinetics

Normoxic studies have reported faster phase II ${\dot{\rm V}}{\rm O}_{2}$ kinetics during the transition from moderate to severe [82], but not unloaded to moderate [55, 82, 83], intensity exercise following NO_3_^−^ supplementation. The apparently greater effects of NO_3_^−^ during the moderate to severe intensity transition might be related to type II muscle fiber-specific effects of NO_3_^−^ [61, 77, 84], given greater relative activation of these fibers during this transition [82]. In hypoxia, ${\dot{\rm V}}{\rm O}_{2}$ kinetics are typically slowed compared with normoxia [36, 85]. Interestingly, Kelly et al. [36] observed faster phase II ${\dot{\rm V}}{\rm O}_{2}$ kinetics during the step transition from unloaded to moderate-intensity but not moderate- to severe-intensity exercise in normobaric hypoxia [36]. Conversely, NO_3_^−^ supplementation did not influence ${\dot{\rm V}}{\rm O}_{2}$ kinetics in normoxia in this study [36]. The precise reasons why NO_3_^−^ had no effect on ${\dot{\rm V}}{\rm O}_{2}$ kinetics during the moderate to severe transition in hypoxia, or either transition in normoxia, are unclear. Nevertheless, the speeding of ${\dot{\rm V}}{\rm O}_{2}$ kinetics during the unloaded to moderate-intensity exercise transition might be beneficial by reducing the time required to reach a steady state and thus minimizing the incurred O_2_ deficit [86].

### Arterial O_2_ Saturation

NO_3_^−^ supplementation has been reported to result in a small (1–4%) increase in arterial O_2_ saturation (S_a_O_2_), as assessed via pulse oximetry, in some [29, 30, 64, 68] but not all [36] studies during acute exposure to normobaric hypoxia. Carriker et al. [65] observed no effects of NO_3_^−^ on S_a_O_2_ during acute exercise in a hypobaric hypoxic chamber (~3500 m).

Only one study has examined the effects of NO_3_^−^ supplementation on S_a_O_2_ at terrestrial altitude [87]. Hennis et al. [87] observed no effect of NO_3_^−^ on S_a_O_2_ during an 11-day trek to Mount Everest Base Camp (~5300 m). The lack of effect in this study may be related to the chronic hypoxic exposure and gradual altitude ascent. Here, endogenous NO generation may be elevated with acclimatization [21], thus potentially diminishing the reliance on exogenous NO_3_^−^ as a substrate for NO generation. NO_3_^−^ might therefore be more effective during acute altitude exposure prior to acclimatization, where NO bioavailability is reduced. However, this awaits direct investigation.

Apnea (i.e., breath holding) investigations provide further insight into the effects of NO_3_^−^ supplementation on S_a_O_2_ when O_2_ availability is restricted. NO_3_^−^ supplementation has been reported to elevate S_a_O_2_ during static [88] and dynamic [89] apneas. Conversely, Schiffer et al. [90] observed lower S_a_O_2_ values and decreased static apnea duration following NO_3_^−^ supplementation. However, NO_3_^−^ supplementation tended to elevate S_a_O_2_ during a maximal dynamic apnea. These conflicting results might be related to methodological differences between studies, including the type of apnea (e.g., static vs. dynamic, underwater vs. ‘dry’), NO_3_^−^ supplementation strategy, and pre-apnea hyperventilation procedures. Nevertheless, the evidence that NO_3_^−^ supplementation can elevate S_a_O_2_ in certain apnea situations, particularly during dynamic apnea, provides support for the increase in S_a_O_2_ observed during exercise in hypoxia [29, 30, 64, 68].

Mechanistically, the elevated S_a_O_2_ reported during exercise in hypoxia following NO_3_^−^ supplementation may be due to reduced muscle O_2_ extraction consequent to improvements in the efficiency of muscle contraction [62] and/or mitochondrial respiration [70]. Additionally, in one study, NO_3_^−^ supplementation increased pulmonary ventilation (${\dot{\rm{V}}}E$) in conjunction with an elevated S_a_O_2_ during exercise in hypoxia [68]. Given a higher ${\dot{\rm{V}}}E$ has previously been associated with reduced arterial desaturation [91], it is possible that an increased ${\dot{\rm{V}}}E$ following NO_3_^−^ supplementation might also play a role in elevating S_a_O_2_. An elevated S_a_O_2_ could have important implications for hypoxic exercise performance, as discussed in Sect. 4. Theoretically, an elevated S_a_O_2_ could also help attenuate acute mountain sickness [92], although current studies have reported no significant effect of NO_3_^−^ on acute mountain sickness [29, 87].

### Tissue Oxygenation

Masschelein et al. [29] reported an increase in the near-infrared spectroscopy (NIRS)-derived tissue oxygenation index (TOI) of the vastus lateralis during cycle ergometry exercise in normobaric hypoxia (F_I_O_2_ 11%, ~5000 m) following NO_3_^−^ supplementation. Kelly et al. [36] also reported a tendency towards elevated TOI during moderate-intensity exercise in normobaric hypoxia (F_I_O_2_ 13.1%, ~3500 m). The TOI reflects the ratio between absolute values of oxygenated and total haemoglobin plus myoglobin, and therefore provides a measure of local oxygenation status. Masschelein et al. [29] interpreted the increased TOI as reflecting a greater O_2_ efficiency consequent to NO_3_^−^. Alternatively, the increased TOI could reflect a greater O_2_ delivery to the muscle due to elevated tissue blood flow [93]. As both hypoxia [94] and NO_3_^−^ [95] have been reported to increase tissue blood flow, this second explanation cannot be excluded. Given the possible influence of skin blood flow (which increases for thermoregulatory purposes during exercise) on the NIRS signal [96], caution is also advised when interpreting these data. Additional information on the strengths and limitations of NIRS is presented elsewhere [93, 97].

### Muscle Metabolism

Exposure to hypoxia reduces both arterial and intracellular PO_2_ [3]. This has a deleterious effect on muscle metabolic function, reducing the capacity for oxidative ATP resynthesis [8, 14], increasing the degradation of limited metabolic substrates including muscle glycogen and PCr and elevating the accumulation of fatigue-associated metabolites such as H^+^, P_i_, and ADP [9–11]. These factors potentially contribute towards the decreased exercise tolerance in hypoxia [98]. Intriguingly, Vanhatalo et al. [11, 99] provide evidence to suggest that NO_3_^−^ supplementation might ameliorate the undesirable effects of hypoxia on muscle metabolic function.

NO_3_^−^ supplementation attenuated the rate of change in muscle [PCr], [P_i_], and pH as assessed via ^31^phosphorus-magnetic resonance spectroscopy (^31^P-MRS) during leg extension exercise in normobaric hypoxia (F_I_O_2_ 14.5%, ~2800 m) [11]. Muscle PCr recovery, reflective of muscle oxidative capacity, was restored to normoxic values. In a follow-up study in more severe hypoxia (F_I_O_2_ 13%, ~3500 m), the faster PCr recovery kinetics with NO_3_^−^ supplementation were associated with an accelerated effective transverse relaxation time (T2*), as obtained via magnetic resonance imaging (MRI) [99]. The T2* signal is a result of both blood flow to the area under investigation and the oxygenation status of the blood and tissue. Consequently, an increase in blood volume and/or the proportion of oxygenated blood increases the T2* signal [100]. The authors speculated that the accelerated T2* recovery following NO_3_^−^ supplementation may indicate a greater delivery of freshly oxygenated blood to the muscle, increasing the O_2_ driving pressure into the muscle cells, and resulting in faster oxidative ATP resynthesis and PCr regeneration [99]. NO_3_^−^ supplementation also improved mitochondrial efficiency, as indicated by an increased muscle phosphorylation potential (indicative of the proton motive force) and lower Gibb’s free energy (∆G) [99]. Interestingly, resting [PCr] was reduced consequent to NO_3_^−^ supplementation, with the magnitude of the decline inversely correlated with plasma [NO_2_^−^]. It was speculated that this might be related to reversible S-nitrosation of creatine kinase (CK), and could serve to enhance mitochondrial sensitivity to ADP stimulation. Thus, NO_3_^−^ supplementation may enhance both muscle energetics and O_2_ delivery during hypoxic exercise, which might partly underpin the improvements in exercise performance discussed in Sect. 4.

### Cardiovascular Response

In normoxia, NO_3_^−^ supplementation has been reported to reduce BP [51–53] and enhance vascular compliance [101] in humans and improve cardiac contractility in rodents [102]. Dietary NO_3_^−^ might also provide a means of altering the complex and multifaceted cardiovascular response to hypoxia.

Some [11, 64], but not all [68, 103], previous studies have reported BP reductions during acute normobaric hypoxic exposure following NO_3_^−^ supplementation. The reduced BP following NO_3_^−^ supplementation has been attributed to increased NO_2_^−^ and NO generation and subsequent vasodilation [52, 53]. Recently, Ingram et al. [104] reported pulmonary and arterial vasodilation after NO_2_^−^ infusion in normobaric hypoxia (F_I_O_2_ 12%, ~4100 m) but not normoxia. The greater activity of NO_2_^−^ reductases, including xanthine oxidoreductase, deoxygenated hemoglobin, and myoglobin, and COX in hypoxia [37] may partly explain this effect. Interestingly, Ingram et al. [104] observed pulmonary vasodilation in hypoxia after plasma [NO_2_^−^] had returned to baseline values. This suggests possible storage and metabolism of NO_2_^−^, and NO in the extravascular tissue could contribute to hypoxia-induced vasodilation [104]. It is possible, based on the findings of Ingram et al. [104], that the capacity to elicit vasodilation and BP effects with NO_3_^−^ supplementation might also be more likely in hypoxia than in normoxia, although direct comparison is required.

In acute normobaric hypoxia (F_I_O_2_ ~11.6%, ~4600 m), NO_3_^−^ supplementation neither influenced the hemodynamic response nor augmented cardiac unloading in healthy men [103]. Similarly, NO_3_^−^ had no effect on the hypoxia-induced hyperemic response to handgrip exercise in young adults (mean ± standard deviation [SD] age 25 ± 1 years) exercising in normoxia and acute normobaric hypoxia (F_I_O_2_ titrated to achieve ~80% S_a_O_2_) [105]. Interestingly, despite similar increases in plasma [NO_2_^−^], NO_3_^−^ supplementation significantly increased the vasodilation and blood flow response to hypoxic exercise in older participants (mean ± SD age 64 ± 2 years). The magnitude of the effect was such that age-related differences in compensatory vasodilation were abolished with NO_3_^−^ supplementation, suggesting therapeutic utility of NO_3_^−^ in older populations exercising in hypoxia [105] or potentially those afflicted with clinical conditions involving tissue hypoxia [106].

Limited evidence is available regarding the effects of NO_3_^−^ on cardiovascular parameters at terrestrial altitude. Bakker et al. [19] and Hennis et al. [87] reported no effects of NO_3_^−^ supplementation on BP during prolonged treks at terrestrial altitude. However, in the study by Bakker et al. [19], acute NO_3_^−^ ingestion offset the decline in endothelial function assessed via flow-mediated vasodilation (FMD) in healthy young participants (mean ± SD age 25 ± 5 years) during an expedition in Nepal (28 days >2500 m, including a peak of 3700 m). FMD has traditionally been viewed as reflecting NO-dependent vasodilation of the smooth muscle in response to acute hyperemia following circulatory occlusion [107, 108]. The hyperemia that manifests following occlusion is believed to increase G-protein expression of phosphokinase A and subsequently increase activity of endothelial NOS (eNOS) [108]. NO is then generated via eNOS catabolism of l-arginine, which ultimately results in smooth muscle relaxation and vasodilation [108]. Based on this conventional view, Bakker et al. [19] primarily attributed the beneficial effects of NO_3_^−^ supplementation on FMD to elevated NO bioavailability via the NO_3_^−^–NO_2_^−^–NO pathway and subsequent effects on smooth muscle. However, there exists considerable debate about the NO-dependency of this technique, with some authors supporting [109–111] and others questioning [112–115] the role of NO in FMD assessment. Further exploration may therefore be warranted to understand the precise mechanisms through which NO_3_^−^ supplementation influences FMD.

## Hypoxic Exercise Performance

Several studies have examined the effects of NO_3_^−^ ingestion on exercise capacity/performance in acute normobaric hypoxia (Table 1). The effects of NO_3_^−^ supplementation on exercise capacity/performance in simulated altitude via hypobaric hypoxia and at terrestrial altitude have yet to be investigated. Vanhatalo et al. [11] were the first to study the effects of NO_3_^−^ supplementation during exercise in normobaric hypoxia (F_I_O_2_ 14.5%, ~2800 m) and observed a 21.4% increase in leg-extension TTE with NO_3_^−^ supplementation. This improvement effectively restored exercise duration to normoxia values. Whilst exhaustive leg-extension exercise is not directly representative of athletic performance, these data suggest that NO_3_^−^ supplementation might offer a means of ameliorating the ergolytic effects of hypoxia on exercise performance.

**Table 1:** The effects of nitrate supplementation on exercise performance in hypoxia

References	Participant characteristics^a^	Supplementation protocol	Performance assessment	Trial results^a^	Cohen’s *d*
Vanhatalo et al. [11]	7 M, 2 F; moderately trained (${\dot{\rm V}}{\rm O}_{2\hbox{max} }$ NS)	750 ml BRJ (9.3 mmol NO_3_^−^) 24 h prior, with last 250 ml dose 150 min pre-exercise	Leg extension TTE (F_I_O_2_ 14.5%, ~2800 m)	Normoxia: 471 ± 200 s Hypoxia (PLA): 393 ± 169 s^b^ Hypoxia (NIT): 477 ± 200 s^c^	Normoxia vs. hypoxia PLA: 0.42 Normoxia vs. hypoxia NIT: 0.03 Hypoxia PLA vs. hypoxia NIT: 0.45
Masschelein et al. [29]	15 M; healthy, physically active (${\dot{\rm V}}{\rm O}_{{2{\rm peak}}}$: 61.7 ± 2.1 ml·kg^−1^·min^−1^)	~500 ml·d^−1^ BRJ (0.7 mmol·kg^−1^·day^−1^/~5 mmol·day^−1^ NO_3_^−^) consumed daily for 6 days, with last 500 ml dose 60–120 min pre-exercise	Incremental cycle ergometry TTE (F_I_O_2_ 11%, ~5000 m)	Normoxia: 888 ± 143 s Hypoxia (PLA): 568 ± 89 s^b^ Hypoxia (NIT): 597 ± 85 s^bc^	Normoxia vs. hypoxia PLA: 2.69 Normoxia vs. hypoxia NIT: 2.47 Hypoxia PLA vs. hypoxia NIT: 0.33
Muggeridge et al. [30]	9 M; trained (${\dot{\rm V}}{\rm O}_{{2{\rm peak}}}$ at simulated altitude: 51.9 ± 5.8 ml·kg^−1^·min^−1^)	70 ml concentrated BRJ (~5 mmol NO_3_^−^) consumed 180 min pre-exercise	16.1 km cycle ergometry TT (F_I_O_2_ 15%, ~2500 m)	Hypoxia (PLA): 1702 ± 45 s Hypoxia (NIT): 1664 ± 42 s^c^	Hypoxia PLA vs. hypoxia NIT: 0.87
Kelly et al. [36]	12 M; physically active (${\dot{\rm V}}{\rm O}_{{2{\rm peak}}}$: 58.3 ± 6.3 ml·kg^−1^·min^−1^)	2 × 70 ml·day^−1^ concentrated BRJ (~8.4 mmol·day^−1^ NO_3_^−^) consumed daily for 3 days, with last 140 ml dose consumed 150 min pre-exercise	Cycle ergometry TTE at 75% between GET and ${\dot{\rm V}}{\rm O}_{{2{\rm peak}}}$ (F_I_O_2_ 13.1%, ~3500 m)	Normoxia (PLA): 431 ± 124 s Normoxia (NIT): 412 ± 139 s Hypoxia (PLA): 197 ± 28 s^b^ Hypoxia (NIT): 214 ± 43 s^bc^	Normoxia PLA vs. normoxia NIT: 0.14 Normoxia PLA vs. hypoxia PLA: 2.60 Normoxia PLA vs. hypoxia NIT: 2.34 Hypoxia PLA vs. hypoxia NIT: 0.47
Arnold et al. [67]	10 M; well-trained (${\dot{\rm V}}{\rm O}_{2\hbox{max} }$: 66 ± 7 ml·kg^−1^·min^−1^)	70 ml concentrated BRJ (~7 mmol NO_3_^−^) consumed 150 min pre-exercise	10,000 m treadmill TT (F_I_O_2_ 15.4%, ~2500 m)	Hypoxia (PLA): 2862 ± 233 s Hypoxic (NIT): 2874 ± 265 s	Hypoxia PLA vs. hypoxia NIT: 0.05
Arnold et al. [67]	10 M; well-trained (${\dot{\rm V}}{\rm O}_{2\hbox{max} }$: 66 ± 7 ml·kg^−1^·min^−1^)	70 ml concentrated BRJ (~7 mmol NO_3_^−^) consumed 150 min pre-exercise	Incremental treadmill TTE (F_I_O_2_ 12.8%, ~4000 m)	Hypoxia (PLA): 393 ± 62 s Hypoxic (NIT): 402 ± 80 s	Hypoxia PLA vs. hypoxia NIT: 0.13
MacLeod et al. [66]	11 M; well-trained (${\dot{\rm V}}{\rm O}_{{2{\rm peak}}}$: 67.5 ± 5.8 ml·kg^−1^·min^−1^)	70 ml concentrated BRJ (~6 mmol NO_3_^−^) consumed 120 min pre-exercise	10 km cycle ergometry TT in normoxia and hypoxia (F_I_O_2_ ~15%, ~2500 m)	Normoxia (PLA): 954 ± 47 s Normoxia (NIT): 961 ± 54 s Hypoxia (PLA): 1023 ± 49 s^b^ Hypoxia (NIT): 1018 ± 52 s^b^	Normoxia PLA vs. normoxia NIT: 0.14 Normoxia PLA vs. hypoxia PLA: 1.44 Normoxia PLA vs. hypoxia NIT: 1.29 Hypoxia PLA vs. hypoxia NIT: 0.10
Bourdillon et al. [68]	12 M; well-trained (${\dot{\rm V}}{\rm O}_{2\hbox{max} }$ NS)	0.1 mmol·kg^−1^·day^−1^ NaNO_3_^−^ for 3 days	15 km cycle ergometry TT in normoxia and hypoxia (F_I_O_2_ 11%, ~5000 m)	Normoxia (PLA): 1597 ± 96 s Normoxia (NIT): 1581 ± 63 s Hypoxia (PLA): 2155 ± 687 s^b^ Hypoxia (NIT): 2005 ± 309 s^b^	Normoxia PLA vs. normoxia NIT: 0.20 Normoxia PLA vs. hypoxia PLA: 1.14 Normoxia PLA vs. hypoxia NIT: 1.78 Hypoxia PLA vs. hypoxia NIT: 0.29
Shannon et al. [64]	12 M; spectrum of untrained to well-trained (${\dot{\rm V}}{\rm O}_{2\hbox{max} }$ 62.1 ± 9.3 ml·kg^−1^·min^−1^)	140 ml concentrated BRJ (15.2 mmol NO_3_^−^) consumed 180 min pre-exercise	1500 m treadmill TT in hypoxia (F_I_O_2_ ~15%, ~2500 m)	Hypoxia (PLA): 342 ± 46 s Hypoxia (NIT): 331 ± 45 s^c^	Hypoxia PLA vs. hypoxia NIT: 0.24

*BRJ* beetroot juice, *F* female, *F*_*I*_ *O*_*2*_ fraction of inspired oxygen, *GET* gas exchange threshold, *M* male, *NIT* nitrate, *NS* not specified, *PLA* placebo, *TT* time-trial, *TTE* time to exhaustion, ${\dot{V}}{O}_{2max }$ maximal oxygen uptake, ${\dot{V}}{O}_{2peak}$ peak oxygen uptake

^a^Values are mean ± standard deviation

^b^Significantly different to normoxia

^c^Significantly different to PLA

Masschelein et al. [29] confirmed the beneficial effects of NO_3_^−^ supplementation in extreme normobaric hypoxia (F_I_O_2_ 11%, ~5000 m). Cycle ergometry TTE was reduced by 36% in hypoxia versus normoxia. However, NO_3_^−^ supplementation attenuated ~5% of this ergolytic effect. The magnitude of this effect is substantially lower than that reported by Vanhatalo et al. [11], although it is likely of practical relevance to an athlete and may be accounted for by methodological differences. Notably, in the study by Vanhatalo et al. [11], exercise was conducted in moderate hypoxia involving a small muscle group, whereas Masschelein et al. [29] had participants complete whole-body exercise in severe hypoxia. In the former, a beneficial effect of NO_3_^−^ on muscle O_2_ efficiency may result in a direct and substantial improvement in performance, given muscles are over perfused and muscle ${\dot{\rm V}}{\rm O}_{2}$ is predominantly limited by mitochondrial O_2_ consumption rather than delivery of O_2_ to the muscle [11]. In the latter, muscle O_2_ consumption is limited primarily by impaired O_2_ diffusion, consequent to the drop in arterial PO_2_, such that greater O_2_ efficiency may result in smaller performance improvements [29]. Additionally, constant-load TTE tests, as employed by Vanhatalo et al. [11], are typically more sensitive than incremental TTE tests for detecting improvements in exercise capacity [29, 116].

Muggeridge et al. [30] first assessed the effects of NO_3_^−^ supplementation on TT performance rather than TTE in normobaric hypoxia (F_I_O_2_ 15%, ~2500 m). Performance in a pre-loaded (i.e., preceded by a steady-state period) 16.1-km cycle ergometry TT was significantly faster (2.2%) following NO_3_^−^ supplementation. Kelly et al. [36] reported similar beneficial effects of NO_3_^−^ on severe-intensity cycle ergometry TTE in physically active males (mean ± SD ${\dot{\rm V}}{\rm O}_{{2{\rm peak}}}$: 58.3 ± 6.3 ml·kg^−1^·min^−1^) exercising in normobaric hypoxia (F_I_O_2_ 13.1%, ~3500 m). Interestingly, NO_3_^−^ supplementation did not influence exercise tolerance in normoxia in this study [36], supporting the notion that the ergogenic effects of NO_3_^−^ might be particularly pronounced in conditions of low O_2_ availability. Most recently, Shannon et al. [64] reported a significant improvement in pre-loaded 1500-m running TT performance in normobaric hypoxia (F_I_O_2_ ~15%, ~2500 m) following NO_3_^−^ supplementation in participants across a range of different fitness levels (${\dot{\rm V}}{\rm O}_{2\hbox{max} }$ range: 47.1–76.8 ml·kg^−1^·min^−1^). All 12 participants in that study were faster with NO_3_^−^ supplementation than with placebo, with an average improvement in 1500 m TT performance of 3.2%.

However, not all studies have observed a positive effect of NO_3_^−^ supplementation on hypoxic exercise performance. MacLeod et al. [66] reported no effects of NO_3_^−^ supplementation on 10-km cycle ergometry TT performance in well-trained athletes (mean ± SD ${\dot{\rm V}}{\rm O}_{{2{\rm peak}}}$ 67.5 ± 5.8 ml·kg^−1^·min^−1^) exercising in either normobaric hypoxia (F_I_O_2_ ~15%, ~2500 m) or normoxia. Likewise, Bourdillon et al. [68] observed no significant difference in 15-km cycle ergometry TT performance in extreme normobaric hypoxia (F_I_O_2_ 11%, ~5000 m) or normoxia following NO_3_^−^ supplementation. However, there was a non-significant tendency towards improved performance (~151 s faster) in hypoxia with NO_3_^−^ ingestion. Similarly, Arnold et al. [67] reported no effect of NO_3_^−^ ingestion on 10-km TT running performance in moderate normobaric hypoxia (F_I_O_2_ 15.4%, ~2500 m) or incremental running performance in extreme normobaric hypoxia (F_I_O_2_ 12.8%, ~4000 m) in highly trained runners (mean ± SD ${\dot{\rm V}}{\rm O}_{2\hbox{max} }$ 66 ± 7 ml·kg^−1^·min^−1^).

These conflicting results might be explained by methodological differences between investigations. At present, some evidence exists to suggest that the training status of participants, exercise protocol, supplementation protocol, and exercise environment (i.e., the degree and duration of hypoxic exposure) can all moderate the effects of NO_3_^−^ supplementation [31]. However, it should be noted that none of these variables alone can entirely explain the apparent ergogenic effect of NO_3_^−^ supplementation (or lack thereof), and it is the interaction between these and other factors that appears to be important.

In several normoxic studies, high aerobic fitness has been suggested as a possible reason for minimal effects of NO_3_^−^ on exercise performance [117–122]. Notably, Porcelli et al. [58] suggested that untrained and moderately trained (${\dot{\rm V}}{\rm O}_{2\hbox{max} }$ <60 ml·kg^−1^·min^−1^) individuals are more likely to benefit from NO_3_^−^ supplementation than endurance-trained athletes (${\dot{\rm V}}{\rm O}_{2\hbox{max} }$ >60 ml·kg^−1^·min^−1^). However, given the cross-sectional nature of this study and relatively modest sample size (fitness-stratified sub-groups of six to eight participants), these data should be interpreted with caution. Moreover, a recent meta-analysis found no influence of aerobic fitness on the response to NO_3_^−^ supplementation [59], bringing into question this relationship. Nevertheless, as evident in Shannon et al. [64], who observed beneficial effects of NO_3_^−^ in participants across a spectrum of fitness levels during exercise in moderate normobaric hypoxia, a high training status does not entirely preclude an ergogenic effect of NO_3_^−^ supplementation. Given that NO_3_^−^ supplementation appears to be more effective in hypoxia than in normoxia [123], it is possible that individuals who may not benefit from NO_3_^−^ supplementation in normoxia (or at low/moderate altitudes) might derive an ergogenic effect in extreme hypoxia where the NO_3_^−^–NO_2_^−^–NO pathway is enhanced yet NOS activity is suppressed.

NO_3_^−^ supplementation appears to be most effective during short-duration high-intensity exercise, although data are presently limited regarding the effects of NO_3_^−^ on this type of exercise at altitude. During short-duration high-intensity activity, the cellular environment is likely to be particularly hypoxic and acidic, potentiating NO_2_^−^ reduction into NO [49, 124]. Moreover, type II muscle is likely to be highly activated, and compelling evidence from murine models suggests that NO_3_^−^ supplementation might solely increase perfusion to [84, 95] and contractile function/force generation of [77] type II muscle. Whilst a precise ‘cut-off’ duration or exercise intensity for the effects of NO_3_^−^ supplementation to be meaningful has yet to be established and will likely differ depending on other experimental factors, it appears that NO_3_^−^ supplementation is usually beneficial for exercise tests lasting <30 min [55, 57, 58, 62, 63, 125, 126], but usually not longer [117, 118, 120, 127], at least in normoxia. Exercise at altitude, particularly mountaineering and hiking, can include sustained periods of relatively low-intensity activity. The effects of NO_3_^−^ supplementation on key physiological and functional (e.g., walking distance in a given time period) parameters during this type of exercise warrant further study, especially as only a small proportion of people ascending to altitude are highly trained athletes conducting high-intensity exercise. Interestingly, Kuennen et al. [128] reported a lower O_2_ cost of exercise during a simulated desert march in the heat (41 °C) consequent to NO_3_^−^ supplementation. Given the similarity of this exercise mode to altitude hiking (i.e., relatively low-intensity walking whilst carrying a backpack), it is possible that NO_3_^−^ supplementation might also reduce O_2_ consumption during altitude hiking, although this has yet to be investigated. The effect of NO_3_^−^ supplementation at lower altitudes (<2500 m >sea level) also remains unexplored yet is of interest given physiological functioning may be influenced by altitudes as low as 300–800 m [4].

Finally, normoxic studies suggest the supplementation strategy might influence the ergogenic effects of NO_3_^−^. First, there is evidence to indicate a possible dose response to NO_3_^−^ supplementation [38, 129, 130]. Likewise, some effects of NO_3_^−^, particularly those requiring changes in protein expression [70, 77], may require several days to occur, suggesting chronic loading may be more effective than acute supplementation [59, 63]. Interestingly, Flueck et al. [81] reported greater reductions in ${\dot{\rm V}}{\rm O}_{2}$ with beetroot juice than with NaNO_3_^−^ supplementation, which could be related to greater NO_2_^−^ reduction into NO with beetroot juice consequent to the polyphenols and/or antioxidants. The potential influence of these moderators awaits investigation in hypoxia.

### Simulated Versus Terrestrial Altitude

To date, few studies have explored the efficacy of NO_3_^−^ supplementation during exposure to terrestrial altitude [19, 87, 131], presumably because of the high financial costs and logistical difficulties associated with such investigations. Instead, researchers have typically administered inspired hypoxic gas during exercise or conducted exercise in normobaric or (to a lesser degree) hypobaric hypoxic chambers. There is ongoing debate about potential differences in the physiological response to normobaric hypoxia and terrestrial altitude [132], including potential disparities in NO metabolism [133]. Further investigations into the effects of NO_3_^−^ at terrestrial altitude and/or contrasting the effects of NO_3_^−^ supplementation between terrestrial and simulated altitude is warranted. It seems likely that NO_3_^−^ supplementation could enhance exercise performance at terrestrial altitude, given exercise performance is predominantly limited by the low PO_2_ in both normobaric and hypobaric hypoxia [29]. Furthermore, NO_3_^−^ supplementation has been reported to improve endothelial function at terrestrial altitude, suggesting the potential to alter peripheral vascular function in this environment [19]. Given the large degree of “noise” associated with field-based testing, future studies may require large sample sizes to ensure sufficient power to detect any meaningful physiological changes with NO_3_^−^ supplementation.

## Training in Hypoxia

There remains a degree of ambiguity over the performance effects of NO_3_^−^ supplementation during exercise in hypoxia, as discussed in Sect. 4. Nevertheless, it is possible that NO_3_^−^ supplementation could help augment the physiological and performance adaptations to training in hypoxia, if the maintainable work rate during training sessions was enhanced or prolonged. Moreover, NO has been implicated in the adaptation to hypoxia [21] and appears to play a role in skeletal muscle hypertrophy and fiber-type transitions [134] and endothelial adaptations [135], but not mitochondrial biogenesis [136, 137], following exercise training. Increasing NO bioavailability via NO_3_^−^ supplementation might therefore enhance some of the adaptations to hypoxic training. Conversely, it is also possible that NO_3_^−^ might suppress hypoxic training adaptations by limiting the drop in arterial and muscle O_2_ saturation, factors that could serve as “signals” for adaptation to hypoxic training [138].

Puype et al. [138] found no effect of NO_3_^−^ supplementation on the physiological and performance adaptations to 6 weeks of high-intensity endurance training (5 × 30 min·week^−1^ cycle ergometry at 4–6 mmol·l^−1^ blood [lactate]) in normobaric hypoxia (F_I_O_2_ 12.5%, ~4000 m). Improvements in ${\dot{\rm V}}{\rm O}_{2\hbox{max} }$, power output at the onset of blood lactate accumulation (OBLA; 4 mmol·l^−1^ blood [lactate]) and average power output during a 30-min TT in normoxia were similar in NO_3_^−^ and placebo conditions. Likewise, the pre- to post-hypoxic training change was similar between groups for muscle adenosine monophosphate-activated kinase (AMPK) protein content and phosphorylation, hypoxia-inducible factor (HIF)-1α messenger RNA (mRNA) content, and glycogen breakdown during the TT. More recently, De Smet et al. [139] explored the effects of NO_3_^−^ on adaptations to sprint interval training (SIT) in normobaric hypoxia (F_i_O_2_ 15%, ~2500 m) versus separate unsupplemented normoxic and hypoxic training groups. Participants completed 3 × 4–6 maximal 30 s cycle ergometry sprints per week over a 5-week period. The proportion of type IIa fibers was significantly increased in the hypoxic NO_3_^−^ group, whereas the proportion of type IIx fibers was significantly decreased. By contrast, a significant decrease in the proportion of type IIx fibers in the unsupplemented groups did not lead to significant increases in other fiber-type groups. These findings were coupled with a tendency towards greater improvements in normoxic 30-s sprint performance in the hypoxic NO_3_^−^ training group relative to the hypoxic unsupplemented training group. Conversely, performance in a 30-min TT completed in normoxia increased by similar amounts in all groups. Taken together, the results of Puype et al. [138] and De Smet et al. [139] suggest that NO_3_^−^ supplementation consumed during 5–6 weeks of endurance or sprint training in hypoxia has minimal effects on physiological adaptations or on exercise performance in normoxia.

Two studies have also recently explored the effects of NO_3_^−^ supplementation on the adaptations to SIT in normoxia [140, 141]. Muggeridge et al. [140] reported greater improvements in maximal work rate during incremental exercise and indices of repeated high-intensity performance in participants who ingested NO_3_^−^ versus placebo prior to SIT sessions (3 × 4–6 maximal 15 s cycle ergometry sprints per week, for 3 weeks). Thompson et al. [141] also demonstrated greater improvements in peak work rate during incremental exercise in individuals who performed SIT (3–4 × 4–5 maximal 30 s cycle ergometry sprints per week for 4 weeks) with NO_3_^−^ compared with both training individuals given a placebo and NO_3_^−^-supplemented individuals who did not train. Interestingly, and supporting the findings of De Smet et al. [139], Thompson et al. [141] showed a decrease in the proportion of type IIx muscle fibers in the vastus lateralis in participants completing SIT with NO_3_^−^ but not in the other conditions. Based on the findings of these studies, it is possible that NO_3_^−^ may elicit similar muscle fiber-type changes when consumed parallel to training in normoxia and hypoxia but may be more effective at enhancing performance adaptations to normoxic than hypoxic training. However, this requires direct investigation given the multifarious methodological differences between studies. In particular, Puype et al. [138] and De Smet et al. [139] in hypoxia recruited moderately trained participants (${\dot{\rm V}}{\rm O}_{2\hbox{max} }$: ~51–60 ml·kg^−1^·min^−1^), whereas Muggeridge et al. [140] and Thompson et al. [141] in normoxia recruited individuals with lower aerobic fitness (${\dot{\rm V}}{\rm O}_{2\hbox{max} }$: ~42–50 ml·kg^−1^·min^−1^), who may exhibit a greater response to training with NO_3_^−^.

The approach adopted in the above studies is similar to most training-intervention studies, whereby a standardized exercise session is utilized throughout the intervention, either with or without an overload component [142–145]. This strategy is seldom adopted by ‘real-world’ athletes, who instead perform a wide variety of training sessions [146–148]. Interestingly, anecdotal reports suggest that some elite endurance athletes at high-altitude training camps selectively use NO_3_^−^ supplements (e.g., beetroot juice) in an attempt to maximize performance in some, but not all, training sessions. This approach, first highlighted by Professor Andrew Jones of Exeter University, UK [31, 32], may allow athletes to benefit from conducting key training sessions at higher exercise intensities. Conversely, low-intensity high-mileage sessions are performed unsupplemented, thus maximizing the hypoxic stimulus for adaptation [31, 32]. Periodizing NO_3_^−^ supplementation in hypoxia has yet to be evaluated scientifically but may offer valuable psychological advantages to athletes regardless of the potential physiological merits.

## Summary and Conclusions

NO_3_^−^ supplementation is emerging as a promising nutritional aid, with potentially beneficial applications for the wide variety of individuals ascending to altitude each year. In this review, NO_3_^−^ supplementation has been demonstrated to reduce pulmonary ${\dot{\rm V}}{\rm O}_{2}$ and, in some cases, elevate S_a_O_2_ in normobaric hypoxia—effects that may be attributable to improvements in the efficiency of muscle contraction and/or mitochondrial respiration, and are of functional relevance for individuals exercising in a low-O_2_ environment. Current evidence also suggests that NO_3_^−^ supplementation can improve muscle energetics during exercise in normobaric hypoxia via effects on tissue O_2_ delivery and consumption and might alter cardiovascular responses to normobaric and hypobaric hypoxia. In contrast, whilst NO_3_^−^ supplementation might influence some of the physiological responses to hypoxic training, current evidence suggests that this does not translate into improved exercise performance in normoxia. Given that the majority of published investigations have explored the effects of NO_3_^−^ supplementation in simulated altitude, which is often regarded as an incomplete surrogate of “true” altitude, future studies at terrestrial altitude are necessary. Researchers are encouraged to further probe the myriad potentially beneficial effects of NO_3_^−^ supplementation as a potential aid to “beet-ing” the mountain.
